# Vascular access infection by *Staphylococcus aureus* from removed dialysis accesses

**DOI:** 10.1002/mbo3.800

**Published:** 2019-01-25

**Authors:** Chishih Chu, Min Yi Wong, Yuan‐Hsi Tseng, Chun‐Liang Lin, Chun‐Wu Tung, Chih‐Chen Kao, Yao‐Kuang Huang

**Affiliations:** ^1^ Department of Microbiology, Immunology, and Biopharmaceuticals National Chiayi University Chiayi City Taiwan (R.O.C.); ^2^ Division of Thoracic and Cardiovascular Surgery Chiayi Chang Gung Memorial Hospital and College of Medicine, Chang Gung University, Taoyuan Puzi City Chiayi County Taiwan (R.O.C.); ^3^ Department of Nephrology Chiayi Chang Gung Memorial Hospital Puzi City Chiayi County Taiwan (R.O.C.)

**Keywords:** antibiotic resistance, hemodialysis, MRSA, MSSA, SCC*mec*, vascular access infection

## Abstract

Hemodialysis patients are particularly vulnerable to *Staphylococcus aureus* infection, with the vascular access serving as the site of entry for this formidable pathogen. Patients with arteriovenous grafts (AVGs) and tunneled‐cuffed catheters (TCCs) are at elevated risk of *S. aureus* infection. In this study, we investigated the correlation between the clinical characteristics of *S. aureus* vascular access infection (VAI), molecular profiles, and the biofilm formation abilities of clinical isolates of *S. aureus*. We collected samples of methicillin‐resistant *S. aureus *(MRSA), methicillin‐sensitive *S. aureus* (MSSA), and methicillin‐sensitive *S. argenteus* (MSSAg) from patients with *S. aureus* VAI and patients with other infections. The molecular profiles of the clinical isolates were determined using disk diffusion testing and molecular typing. The biofilm formation ability was determined by microtiter plate assay. In total, 63 *S. aureus* and 10 *S. argenteus* isolates were identified: 40 MRSA, 23 MSSA, and ten MSSAg. MRSA was highly prevalent (77.8%) in TCC isolates and was multidrug resistant. Of the 40 MRSA isolates, ST239‐SCC*mec* III was the predominant clone. SCC*mec* type IV was the predominant type (35%) in isolates from AVGs, while SCC*mec* type III was prevalent in TCC infection and showed significantly higher biofilm formation ability than types IV and V. In dialysis VAI by *S. aureus*, patients with TCC were more often infected with MRSA than patients with AVG, and MRSA in TCC–VAI was predominantly SCC*mec* type III, which had the strongest drug resistance and biofilm formation ability.

## 1 BACKGROUND

Hemodialysis requires proper and durable vascular access that can tolerate high blood flow between the patient's circulation and the dialyzer during hemodialysis. Arteriovenous fistulae, arteriovenous grafts (AVGs), and tunneled‐cuffed catheters (TCCs) are among the most common vascular access choices. Vascular access infection (VAI) is a major complication in patients undergoing renal replacement therapy and results in vascular access loss, long hospital stays, and mortality. Vascular access for hemodialysis is particularly vulnerable to *Staphylococcus aureus *(*S. aureus*) infection (Vandecasteele, Boelaert, & Vriese, 2009). *S. aureus* is a common human pathogen responsible for a wide range of clinical infections, such as bacteremia, as well as infective endocarditis (IE) and osteoarticular, skin, soft tissue, and device‐related infection (Tong, Davis, Eichenberger, Holland, & Fowler, 2015). *S. aureus* can survive under extreme conditions and form long‐lasting colonization in human tissue or biofilms on the surfaces of foreign devices. The biofilm is produced by a combination of host factors (e.g., fibrinogen, fibrin, fibronectin, and extracellular polysaccharides) and microbial products (e.g., glycocalyx or “slime”) and has a critical role in bacterial antimicrobial resistance and recalcitrant infection.

Microbial infection with multidrug‐resistant microorganisms poses an increasing and critical public health problem worldwide. Methicillin‐resistant *S. aureus* (MRSA) has antimicrobial resistance to all penicillins including methicillin. MRSA strains are a leading cause of healthcare‐associated infection as well as a major cause of community‐acquired infection worldwide. A mobile genetic element—staphylococcal cassette chromosome *mec* (SCC*mec*)—is a feature used for MRSA classification. The SCC*mec* element carries the methicillin resistance determinant *mecA* and its regulatory genes (International Working Group on the Classification of Staphylococcal Cassette Chromosome, 2009). SCC*mec *type I to III elements, which are responsible for resistance to numerous classes of antibiotics are found in hospital‐associated MRSA (HA‐MRSA) strains, whereas the SCC*mec *type IV and V elements are commonly found in community‐associated MRSA (CA‐MRSA) strains (Kang et al., 2015).

In this study, we investigated the association between the clinical characteristics of *S. aureus* VAI, microbial features (including epidemiologic and SCC*mec *classification)*,* and the biofilm formation ability of *S. aureus *clinical isolates from surgical specimens.

## 2 MATERIALS AND METHODS

### 2.1 Patient characteristics

This study was performed at a territory referral hospital in Taiwan between September 2013 and December 2016. We prospectively collected information on 33 consecutive patients with *S. aureus *VAI who required removal of AVGs and TCCs. In addition, 14 patients hospitalized between September 2013 and December 2016 with *S. aureus *infection from diseases other than VAI including empyema, port‐A catheter infection, chronic ischemic leg, infected endocarditis, and other surgical wound infections were also analyzed for comparison. Patients with poor compliance and those who declined to be part of this study were excluded. All patients were provided explanations about the procedures individually, and informed consent was obtained prior to performing the procedures.

According to the Centers for Disease Control and Prevention (CDC), the epidemiologic definition of healthcare‐associated infection is a set of infections in which the patients had hospitalization, surgery, dialysis, residence in a long‐term care facility, or usage of indwelling catheters in the preceding 12 months. Typically, patients undergoing renal replacement therapies return to community dialysis clinics after their acute illnesses have stabilized. To discuss the possible source of VAI in patients with hemodialysis, the isolates were classified as healthcare‐associated hospital‐onset (HAHO) if the culture was obtained >3 calendar days after a hospital admission (admission day is considered day 1) or healthcare‐associated community‐onset (HACO) if the culture was obtained as outpatient or ≤3 calendar days after a hospital admission with major healthcare risk factors (dialysis, hospitalization, surgery, or long‐term care residence within 1 year prior to collection of the positive MRSA culture or presence of central venous catheter within 2 days prior) (Gualandi et al., 2018; Nguyen et al., 2013).

In all cases of hemodialysis VAI, antibiotic therapy was initiated with broad‐spectrum antibiotics. Based on the results of the culture and sensitivities, conversion to an appropriate antibiotic was indicated. The infected hemodialysis vascular accesses were removed in the event of uncontrolled access‐related bacteremia and severe complications (progressing to pseudoaneurysms or necrotizing fasciitis).

### 2.2 Microbiological investigations

73 bacterial isolates from 47 patients’ blood and contaminated device samples were cultured under laboratory standards. The samples were routinely cultured on blood agar at 37°C overnight. Strain identification was performed using standard biochemical (phenotypic) procedures using bioMérieux's API Staph system.

### 2.3 Antibiotic susceptibility testing

The antimicrobial susceptibility of *S. aureus* was determined by the disk diffusion method on Mueller–Hinton agar using the following antibiotics: clindamycin, erythromycin, fusidic acid, oxacillin, penicillin, trimethoprim‐sulfamethoxazole, and tigecycline in accordance with the standard of the Clinical and Laboratory Standards Institute (CLSI) (CLSI, 2013).

### 2.4 Genomic DNA extraction

A single colony from a *S. aureus* isolate and from a *S. argenteus* isolate were inoculated in brain heart infusion (BHI) broth for 16 hr and 1 ml of overnight culture was harvested using centrifugation at 16,500 x *g* for 5 min. Bacterial cells were suspended in 1 ml of ultrapure water and heated at 100°C for 15 min. The supernatant containing the DNA was stored at 4°C until further use.

### 2.5 Molecular characterization

#### 2.5.1 *Spa* typing, multilocus sequence typing (MLST), SCC*mec* typing and *mecA* gene confirmation

Of 73 isolates, the staphylococcal protein A gene polymorphic region (*spa*) was amplified using the primer pairs as described previously and sequenced (Strommenger et al., 2008). The *spa* types were assigned using the BioNumerics software package (Applied Maths, Sint‐Martens‐Latem, Belgium). Multilocus sequencing typing (MLST) was conducted as described previously (Enright, Day, Davies, Peacock, & Spratt, 2000). The allele numbers and sequence types (STs) were assigned according to the *S. aureus* MLST database (https://pubmlst.org/saureus/). To confirm MRSA, *mecA* gene detection was performed using PCR as described previously (Pournajaf et al., 2014). Multiplex PCR with four primer pairs was performed to identify the SCC*mec* types I to V (Boye, Bartels, Andersen, Moller, & Westh, 2007).

#### 2.5.2 Erythromycin resistance gene detection

The presence of several antibiotic resistance genes was identified using multiplex PCR with the 16S rDNA gene as an internal control. Presence of the following genes conferring resistance to erythromycin was tested: *erm*(A), *erm*(B), *erm*(C), and *msr*(A) (Martineau et al., 2000; Strommenger, Kettlitz, Werner, & Witte, 2003).

### 2.6 Biofilm formation assay

The biofilm production of *S. aureus* isolates at different timepoints was determined quantitatively by using a modified microtiter plate assay as described previously (Stepanović, Vuković, Dakić, Savić, & Švabić‐Vlahović, 2000). The bacterial isolates were grown in tryptic soy broth (TSB) supplemented with 0.25% glucose at 37°C overnight. Flat‐bottomed plastic microtiter plates were filled with 200 μl of 1:40 diluted cultures and incubated for 4, 8, and 24 hr at 37°C. The negative control wells contained only broth. A known biofilm‐forming *S. aureus* reference strain, ATCC 29213, was used as the positive control. Wells were gently washed with sterile physiological saline, fixed using 99% methanol, and dried at room temperature. The adherent cells were stained with 0.1% crystal violet, and the bound dye was dissolved using 33% glacial acetic acid per well. Finally, the optical density (OD) of each well was measured at 570 nm using an ELISA reader. Based on the obtained OD value, the strength of biofilm formation was calculated according to the standard Christensen et al. (1985) cut‐off value classification formula as follows: optical density cut‐off value (ODc) = average OD of negative control +3 × standard deviation (*SD*) of negative control. Strains were classified as follows: OD ≤ ODc nonadherent; ODc <OD ≤ 2 × ODc weakly adherent; 2 × ODc < OD ≤ 4 × ODc moderately adherent; and 4 × ODc < OD strongly adherent.

### 2.7 Statistical analysis

Categorical variables were compared using the chi‐square (χ^2^) test of IBM SPSS Statistics for Windows, Version 22.0 (Armonk, NY: IBM Corp.). Statistical analysis of the biofilm formation was performed using GraphPad Prism version 5.01 for Windows (GraphPad Software, La Jolla, California, USA, www.graphpad.com). Data were analyzed using one‐way ANOVA followed by Bonferroni's multiple comparison tests. All statistical analyses were conducted using STATA statistics/Data Analysis 8.0 software (Stata Corporation, College Station, TX). A *p < *0.05 was considered statistically significant.

## 3 RESULTS

### 3.1 Patient characteristics

From 2013 to 2016, 42 patients with *S. aureus *infection and five patients with *S. argenteus* infection were enrolled in this study. The *S. aureus* infection group included 17 patients with infected AVGs, 11 patients with infected TCCs, and 14 patients with other sources of *S. aureus *infection. The *S. argenteus* infection group included three patients with infected AVGs and two patients with infected TCCs. The characteristics describing this population are listed in Table 1. The patients in the AVG and TCC groups were more diabetic than other groups. The patients with other sources of infection had similar comorbidities as AVG and TCC groups except diabetic.

**Table 1:** Clinical characteristics of patients with *S. aureus* and *S. argenteus* isolates

Clinical characteristic	AVG (*n* = 20)			TCCs (*n* = 13)			Others (*n* = 14)	
	MRSA (*n* = 9)	MSSA (*n* = 8)	MSSAg (*n* = 3)	MRSA (*n* = 10)	MSSA (*n* = 1)	MSSAg (*n* = 2)	MRSA (*n* = 8)	MSSA (*n* = 6)
Gender								
Male (*n* = 25)	3 (33.3)	2 (25)	1 (33.3)	6 (60)	1 (100)	0 (0)	6 (75)	6 (100)
Female (*n* = 22)	6 (66.7)	6 (75)	2 (66.7)	4 (40)	0 (0)	2 (100)	2 (25)	0 (0)
Age (year)	72.1	68.5	61.9	62.81	62.4	60.7	64.95	59.01
Comorbidity								
HTN	9 (100)	6 (75)	2 (66.7)	9 (90)	1 (100)	2 (100)	6 (75)	4 (66.7)
ESRD	9 (100)	8 (100)	3 (100)	10 (100)	1 (100)	2 (100)	2 (25)	3 (50)
DM	8 (88.9)	4 (50)	3 (100)	7 (70)	0 (0)	2 (100)	3 (37.5)	3 (50)
Renal insufficiency	0 (0)	0 (0)	0 (0)	0 (0)	0 (0)	0 (0)	1 (12.5)	0 (0)
Hepatitis B	0 (0)	0 (0)	0 (0)	1 (10)	1 (100)	0 (0)	3 (37.5)	0 (0)
Hepatitis C	3 (33.3)	2 (25)	0 (0)	2 (20)	0 (0)	0 (0)	1 (12.5)	0 (0)
Gouty arthritis	3 (33.3)	0 (0)	0 (0)	0 (0)	1 (100)	0 (0)	0 (0)	0 (0)
Mental retardation	0 (0)	0 (0)	0 (0)	0 (0)	0 (0)	0 (0)	1 (12.5)	0 (0)
CAD	1 (11.1)	0 (0)	0 (0)	2 (20)	0 (0)	0 (0)	2 (25)	2 (33.3)
Atrial fibrillation	1 (11.1)	0 (0)	0 (0)	1 (10)	0 (0)	0 (0)	0 (0)	1 (16.7)
CHF	3 (33.3)	0 (0)	0 (0)	0 (0)	0 (0)	0 (0)	1 (12.5)	1 (16.7)
Colon cancer	0 (0)	1 (12.5)	0 (0)	1 (10)	0 (0)	0 (0)	0 (0)	0 (0)

Note

MRSA: methicillin‐resistant *S. aureus*; MSSA: methicillin‐sensitive *S. aureus*; MSSAg: methicillin‐sensitive *S. argenteus*; HTN: hypertension; ESRD: endstage renal disease; DM: diabetes mellitus; CAD: coronary artery disease; CHF: congestive heart failure; AVG: arteriovenous graft. TCCs: tunneled‐cuffed catheters; others included empyema, port‐A catheter infection, chronic ischemic leg, infected endocarditis, and surgical wound infections.

### 3.2 Antimicrobial susceptibility testing

We collected a total of 39 *S. aureus* isolates from 28 hemodialysis patients (AVGs and TCCs) as well as 24 isolates from 14 patients with other diseases or surgical infections. In addition, ten *S. argenteus* isolates were collected from five hemodialysis patients. The isolates were confirmed to be MRSA with oxacillin resistance by susceptibility testing and were confirmed to be *mecA *positive by PCR. Of the 73 isolates, 40 (54.8%) were MRSA, 23 (31.5%) were MSSA isolates, and 10 (13.7%) were methicillin‐sensitive *S. argenteus* (MSSAg). The isolates from the TCCs showed a higher percentage of MRSA (77.78%, 14/18) than the AVG isolates (38.71%, 12/31) and the other sources (58.33%, 14/24) of *S. aureus *infection. A majority of the *S. aureus* isolates were resistant to penicillin (98.41%, 62/63); however, only 50% of the *S. argenteus* isolates showed penicillin resistance. Resistance to non‐β‐lactam antibiotics including clindamycin, fusidic acid, trimethoprim‐sulfamethoxazole (SXT), and tigecycline was higher among the TCCs and other category MRSA isolates. In addition, all of the TCC MRSA isolates (100%, 14/14) were multidrug resistant (≥3 antimicrobial classes)— resistant to at least clindamycin, erythromycin, oxacillin, and penicillin. Meanwhile, 66.7% (8/12) of the AVG MRSA isolates and 78.6% (11/14) of the other MRSA isolates were multidrug resistant. All MRSA isolates were resistant to penicillin and oxacillin except one *mecA*‐positive AVG MRSA isolate that was sensitive to oxacillin. The oxacillin‐resistant *mecA*‐negative isolate was categorized into MRSA. Compared to MRSA, MSSA and MSSAg isolates showed weaker antibiotic resistance, and most of them were resistant only to penicillin.

### 3.3 SCC*mec* typing

The SCC*mec* type classification of the 40 MRSA isolates is shown in Table 2. The *mecA* gene was identified in 97.5% (39/40) of MRSA isolates. The SCC*mec* elements have been classified into the types based on their highly diverse structural organization and genetic content. In this study, four SCC*mec *types (types II, III, IV and V) were found among the 40 MRSA isolates. Of the MRSA isolates, 39.1% (9/23) and 34.8% (8/23) of HAHO‐MRSA carried SCC*mec* types III and IV, respectively, whereas 35.3% (6/17) of HACO‐MRSA carried SCC*mec* types IV and V. The small‐sized SCC*mec* type IV was the most predominant type (35%, 14/40) and was most prevalent in AVG isolates (57.1%, 8/14) followed by type III (30%, 12/40), which was prevalent in TCCs (41.7%, 5/12) and other (58.3%, 7/12) MRSA isolates (Table 3). Collectively, AVG isolates showed similar proportions of MRSA and MSSA (38.71%, 12/31). In addition, AVG MRSA isolates predominantly carried the SCC*mec* IV element (66.7%, 8/12). In contrast, most of the TCC isolates were MRSA (14/18, 77.8%), which predominantly carried SCC*mec* type III and V elements (35.7%, 5/14, respectively).

**Table 2:** Molecular characterization of MRSA isolates

Characteristics	SCC*mec* types				
	II (*n* = 5)	III (*n* = 12)	IV (*n* = 14)	V (*n* = 8)	ND (*n* = 1)
Origin					
AVGs	1 (20)	0 (0)	8 (57.14)	2 (25)	1 (100)
TCCs	2 (40)	5 (41.67)	2 (14.29)	5 (62.5)	0 (0)
Others	2 (40)	7 (58.33)	4 (28.57)	1 (12.5)	0 (0)
MRSA type					
HAHO (*n* = 23)	4 (80)	9 (75)	8 (57.14)	2 (25)	0 (0)
HACO (*n* = 17)	1 (20)	3 (25)	6 (42.86)	6 (75)	1 (100)
ST type					
5	4 (80)	0 (0)	0 (0)	0 (0)	0 (0)
8	0 (0)	0 (0)	1 (7.14)	0 (0)	0 (0)
30	0 (0)	0 (0)	6 (42.86)	0 (0)	0 (0)
45	1 (20)	0 (0)	2 (14.29)	5 (62.5)	0 (0)
59	0 (0)	0 (0)	5 (35.71)	3 (37.5)	0 (0)
239	0 (0)	11 (91.67)	0 (0)	0 (0)	0 (0)
508	0 (0)	0 (0)	0 (0)	0 (0)	1
4,798	0 (0)	1 (8.33)	0 (0)	0 (0)	0 (0)
Antibiotic agents					
Clindamycin	5 (100)	12 (100)^c^	9 (64.3)a,b	7 (87.5)	0 (0)
Erythromycin	5 (100)	12 (100)	11 (78.6)	7 (87.5)	0 (0)
Fusidic acid	2 (40)	10 (83.3)^c^	1 (7.1)^b^	4 (50)a,c	0 (0)
Oxacillin	5 (100)	12 (100)	13 (92.9)	8 (100)	1 (100)
Penicillin	5 (100)	12 (100)	14 (100)	8 (100)	1 (100)
SXT	0 (0)	11 (91.7)^b^	1 (7.1)^a^	0 (0)^a^	0 (0)
Tigecycline	0 (0)	1 (8.3)	0 (0)	0 (0)	0 (0)
Erythromycin resistance genes					
*erm*(A)+*erm*(C)	5	—	—	—	—
*erm*(A)	—	12	—	—	—
*erm*(B)	—	—	5	3	—
*erm*(C)	—	—	5	4	—
*msr*(A)	—	—	1	—	—

Note

AVG: arteriovenous graft; TCCs: tunneled‐cuffed catheters; others included empyema, port‐A catheter infection, chronic ischemic leg, infected endocarditis, and surgical wound infections; HAHO: healthcare‐associated hospital‐onset; HACO: healthcare‐associated community‐onset; SXT: trimethoprim‐sulfamethoxazole

^a‐c^indicate significant differences between different SCC*mec* type for the same antibiotic resistance.

**Table 3:** Epidemiological and genetic analysis of *S. aureus* and *S. argenteus* isolates from different cases

Cases (*n* = 73)	Epidemiological classification, *n* (% within cases)				ST types			Genetic classification, *n* (% within MRSA cases)				
	HAHO‐MRSA	HACO‐MRSA	MSSA	MSSAg	MRSA	MSSA	MSSAg	SCC*mec* II	SCC*mec* III	SCC*mec* IV	SCC*mec* V	ND
AVG (*n* = 31)	6 (19.4%, 6/31)	6 (19.4%, 6/31)	12 (38.7%, 12/31)	7 (22.6%, 7/31)	5, 8, 30, 45, 59, 508	7, 8, 15, 97, 188, 845	2,250	1 (8.3%, 5/12)	0 (0%, 0/12) a	8 (66.7%, 8/12) a	2 (16.7%, 2/12)	1 (8.3%, 1/12)
TCCs (*n* = 18)	7 (38.9%, 7/18)	7 (38.9%, 7/18)	1 (5.6%, 1/18)	3 (16.7%, 3/18)	5, 30, 45, 59, 239, 4,798	7	2,250	2 (14.3%, 5/14)	5 (35.7%, 5/14) b	2 (14.3%, 2/14) b	5 (35.7%, 5/14)	0 (0%)
Others (*n* = 24)	10 (41.7%, 10/24)	4 (16.7%, 4/24)	10 (41.7%, 10/24)	0 (0%)	5, 30 45, 59, 239	7, 15, 97, 3,553, 4,797	‐	2 (14.3%, 5/14)	7 (50%, 7/14) b	4 (28.6%, 4/14) a,b	1 (7.1%, 1/14)	0 (0%)

Note

AVG: arteriovenous graft; TCCs: tunneled‐cuffed catheters; others included empyema, port‐A catheter infection, chronic ischemic leg, infected endocarditis, and surgical wound infections; HAHO: healthcare‐associated hospital‐onset; HACO: healthcare‐associated community‐onset; MRSA: methicillin‐resistant *S. aureus*; MSSA: methicillin‐sensitive *S*. aureus; MSSAg: methicillin‐sensitive *S*. argenteus; ND: not detected

^a‐b^ Indicate significant differences between cases for the same SCC*mec* type.

The resistance profiles of the MRSA isolates differed among the various SCC*mec* types (Tables 2). SCC*mec *type III isolates displayed a high resistance rate (>80%) to six of the tested antibiotics except for tigecycline (8.3%, 1/12). Moreover, the resistance rate of SCC*mec* type III isolates to SXT was significantly higher than that of other SCC*mec* type isolates. All of the SCC*mec *type II isolates were resistant to clindamycin, erythromycin, oxacillin, and penicillin. 87.5% of the SCC*mec *type V isolates were resistant to clindamycin and erythromycin as well as 100% were resistant to oxacillin and penicillin. SCC*mec *type IV, which is prevalent in AVG isolates was predominantly resistant to β‐lactams including penicillin (100%, 14/14) and oxacillin (92.9%, 13/14).

Moreover, the macrolide resistance genes that demonstrated resistance to erythromycin, including *erm*(A), *erm*(B), *erm*(C), and *msr*(A)*,* were prevalent in 58.73% (37/63) of the *S. aureus* isolates and mainly occurred among MRSA isolates (Table 2). Within the erythromycin‐resistant *S. aureus* collection, the *erm*(A) gene was predominant (45.95%, 17/37) followed by the *erm*(C) gene (37.84%, 14/37). In addition, the combination of *erm*(A) and *erm*(C) was found in 13.51% (5/37) of the erythromycin‐resistant *S. aureus* isolates and occurred only in SCC*mec* type II MRSA isolates. Whereas the *erm*(A) gene was mainly found in SCC*mec* type III MRSA isolates, *erm*(B) and *erm*(C) genes were found in SCC*mec* type IV and V isolates. Macrolide resistance by efflux due to the *msr*(A) gene was found in MSSA isolates and one *mecA*‐positive MRSA isolate that was sensitive to oxacillin.

### 3.4 Genetic background characterization

To characterize the genetic background of isolates, all *S. aureus* and *S. argenteus* isolates were typed by the *spa* and MLST approaches (Tables 4 and 5). Of the 63 *S. aureus* isolates, 14 different STs were identified via MLST, among which the most frequently represented were ST239 (*n* = 11), ST45 (*n* = 8), ST59 (*n* = 8), and ST30 (*n* = 6) in 40 MRSA isolates and ST15 (*n* = 9), ST7 (*n* = 4), and ST97 (*n* = 4) in 23 MSSA isolates. The only ST type found in MSSAg was ST2250. Meanwhile, 24 *spa* types were identified. t037 (*n* = 10), t019 (*n* = 5), t002 (*n* = 5), and t1081 (*n* = 5) were predominant in MRSA isolates, whereas t091 (*n* = 4) and t084 (*n* = 4) were predominant in MSSA isolates. However, *spa* typing for all MSSAg isolates was unsuccessful.

**Table 4:** Correlation of ST type, *spa* type, SCC*mec* and antibiotic resistance with MRSA isolates

ST type	*Spa* type	SCC*mec* type	Antibiotic resistance profile	Erythromycin resistance genes	No.
5 (*n* = 4)	t002	II	CLI, ERY, OXA, PEN	*erm*(A)+*erm*(C)	3
			CLI, ERY, FUS, OXA, PEN		1
8 (*n* = 1)	t008	IV	ERY, PEN	*msr*(A)	1
30 (*n* = 6)	t019	IV	CLI, ERY, OXA, PEN	*erm*(C)	3
			ERY, OXA, PEN		1
			OXA, PEN	ND	1
	t1836		OXA, PEN		1
45 (*n* = 8)	t002	II	CLI, ERY, FUS, OXA, PEN	*erm*(A)+*erm*(C)	1
	t026	IV	OXA, PEN	ND	1
	t2383	IV	CLI, ERY, OXA, PEN	*erm*(C)	1
	t1081	V	CLI, ERY, FUS, OXA, PEN		4
			OXA, PEN	ND	1
59 (*n* = 8)	t1751	IV	CLI, ERY, FUS, OXA, PEN, SXT	*erm*(B)	1
	Unknown	IV	CLI, ERY, OXA, PEN		1
	t437	IV	CLI, ERY, OXA, PEN		3
		V	CLI, ERY, OXA, PEN		1
	t3527	V	CLI, ERY, OXA, PEN		2
239 (*n* = 11)	t037	III	CLI, ERY, OXA, PEN, SXT	*erm*(A)	1
			CLI, ERY, FUS, OXA, PEN, SXT		8
	t3528		CLI, ERY, OXA, PEN, SXT, TGC		1
	t748		CLI, ERY, FUS, OXA, PEN		1
508 (*n* = 1)	t026	ND	OXA, PEN	ND	1
4,798 (*n* = 1)	t037	III	CLI, ERY, FUS, OXA, PEN, SXT	*erm*(A)	1

Note

ND: not detected; CLI: clindamycin; ERY: erythromycin; OXA: oxacillin; PEN: penicillin; FUS: fusidic acid; SXT: trimethoprim‐sulfamethoxazole; TGC: tigecycline

**Table 5:** Correlation of ST type, *spa* type, and antibiotic resistance with methicillin‐sensitive *S. aureus* and *S. argenteus* isolates

Strain	ST type	*Spa* type	Antibiotic resistance profile	Erythromycin resistance genes	No.
MSSA (*n* = 23)	7	t091	PEN	none	3
			ERY, PEN	*msr*(A)	1
	8	t008	PEN	none	1
	15	t084	PEN	none	2
		t085	PEN		1
			ERY, PEN	*msr*(A)	1
		t279	PEN	none	2
		t547			1
		t803			2
	97	t224	PEN	none	1
		t267			3
	188	t2769	PEN	none	1
	508	t073	none	none	1
	845	t084	PEN	none	2
	4,797	t213	PEN	none	1
MSSAg (*n* = 10)	2,250	ND	PEN	none	5
			none		5

Note

MSSA: methicillin‐sensitive *S. aureus*; MSSAg: methicillin‐sensitive *S. argenteus*; ND: not detected; ERY: erythromycin; PEN: penicillin

MLST and *spa* types were highly associated with SCC*mec* type in this study. By connecting ST and *spa* with SCC*mec* types, the major clonal distribution of MRSA was ST5‐SCC*mec*II‐t002 (abbreviated as ST5‐II‐t002) (80%, 4/5), ST239‐III‐t037 (75%, 9/12), ST30‐IV‐t019 (35.71%, 5/14), and ST45‐V‐t081 (62.5%, 5/8). However, some ST types were associated with more than one SCC*mec* type. For instance, ST59 isolates carried SCC*mec* type IV and V elements, whereas ST45 isolates carried SCC*mec* type II, IV, and V elements.

### 3.5 Biofilm formation ability

**Figure 1:**
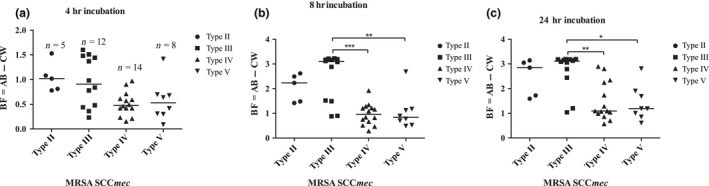
Biofilm formation of MRSA clinical isolates with different SCC*mec* types. The biofilm formation of *mecA*‐positive MRSA isolates (*n* = 39) was investigated after (a) 4 hr, (b) 8 hr, and (c) 24 hr incubation in a microtiter plate. Each solid shape (●■▲▼) represents individual isolates categorized into different SCC*mec* types, and bars indicate the means (*, *p* < 0.05; **, *p* < 0.01; ***, *p* < 0.001; Bonferroni's multiple comparison test following one‐way ANOVA)

To assess the biofilm formation ability of *S. aureus* isolates, we conducted a biofilm formation assay in microtiter plates and incubated for 4, 8, and 24 hr. All of the isolates in this study were biofilm producers with strong adherence after 24 hr. In addition, the biofilm formation ability of MRSA isolates differed among the various SCC*mec* types (Figure 1). The HAHO‐MRSA isolate types II and III showed similar biofilm formation; however, the HAHO‐MRSA type III isolates produced significantly higher biofilms than type IV and V isolates after 4 hr (type IV, *p* < 0.05), 8 hr (both, *p* < 0.001), and 24 hr (both, *p* < 0.01) of incubation.

## 4 DISCUSSION

The higher incidence of infection in the hemodialysis population is because of impaired host immunity due to uremia‐related neutrophil dysfunction and the hemodialysis procedure itself (inadequate water, unfamiliarity with dialyzer use, and repetitive break of skin integrity) (Jaber, 2005). *S. aureus* was present in 2.0% to 68.6% of the isolates, and other gram‐positive bacteria were present in 19.4% to 62.0% of the isolates from patients with VAIs (Lafrance, Rahme, Lelorier, & Iqbal, 2008). *S. aureus *infection is common in the hemodialysis population, which showed a 100‐fold greater incidence than the general population (Centers for Disease Control & Prevention, 2007). *S. aureus* bacteremia and the associated hospital mortality in hemodialysis patients are of clinical importance and have been well documented (Centers for Disease Control & Prevention, 2007; Imaizumi et al., 2017). Previous studies have concluded that *S. aureus* was the leading cause of bloodstream infection and hospital mortality among hemodialysis patients.

VAI by *S. aureus* is one of the worst clinical scenarios in patients undergoing dialysis. VAI is not only potentially lethal but also can result in vascular access loss despite the timely surgical removal of the infected grafts and catheters. Although there have been extensive discussions about *S. aureus* infections in patients with hemodialysis, very few studies have focused on *S. aureus* VAIs and their advanced microbiologic analysis. In this study, we evaluated *S. aureus* from removed infected vascular accesses and analyzed the molecular characteristics, antibiotic resistance patterns, and biofilm formation abilities of the clinical isolates and compared those data with data from *S. aureus* isolates from other surgical infections.

Our data revealed that different antibiotic resistance patterns were related to the use of different vascular accesses (TCC and AVG) in hemodialysis patients. *S. aureus* from TCCs and AVGs belonged to different SCC*mec* types; however, they had similar biofilm formation abilities. *S. aureus* from infected AVGs had a higher percentage of MSSA infection (61.29%, 19/31) than that from TCCs (22.22%, 4/18) and other surgical specimens (41.67%, 10/24). MRSA was dominant in isolates from TCCs. Moreover, all TCC MRSA isolates were multidrug resistant. Most patients in our institution initiated their hemodialysis through TCCs due to their fluid overload/severe azotemia and had subsequent scheduled AVG or arteriovenous fistula creation once stabilized on renal replacement therapy. The patients receiving hemodialysis through TCCs stayed longer in the hospital and had a higher risk of MRSA infection than patients with AVGs.

Previous studies have shown that SCC*mec* type III elements are generally carried by HA‐MRSA, whereas SCC*mec* IV and V are associated with CA‐MRSA (Hiramatsu, Katayama, Yuzawa, & Ito, 2002; Huang & Chen, 2011). In addition, livestock‐associated MRSA (LA‐MRSA) was limited mainly to SCC*mec* type IV and V, which originated from human‐associated MSSA receiving the SCC*mec* element (Price et al., 2012). Nevertheless, in our study, HAHO‐MRSA was discovered to carry type III as well as type IV elements, whereas HACO‐MRSA isolates had both type IV and type V elements with type IV elements predominating. Our observation is in line with the finding of Wang et al. (2015), in which SCC*mec* type IV isolates accounted for HACO‐ and HAHO‐MRSA infections. The increasing incidence of SCC*mec *IV HA‐MRSA in Taiwan has been reported previously (Chen, Huang, Chiu, Su, & Lin, 2005; Huang et al., 2007). This increase indicates that CA‐MRSA is spreading into the hospital setting and may be replacing the conventional HA‐MRSA strains, which have significant clinical and public health implications (Otter & French, 2011, 2012) resulting in the occurrence of HAHO‐ and HACO‐MRSA, which carry SCC*mec* type IV. In this study, SCC*mec* type III was shown to be predominantly carried by TCCs and other surgical specimen MRSA isolates, whereas SCC*mec* type IV was predominant in AVG isolates. These results indicate that the patients receiving hemodialysis through AVGs stay for relatively short durations in the hospital setting and are thus infected by the “community‐type” MRSA (SCC*mec* type IV).

In our findings, different SCC*mec*‐type MRSA with drug resistance varied depending on the specific antibiotic. The SCC*mec* type II and III isolates were multidrug resistant—predominantly to clindamycin, erythromycin, oxacillin, and penicillin, as well as highly resistant to fusidic acid and SXT. In addition, most of the HACO‐MRSA‐associated SCC*mec* type IV and V isolates remained susceptible to SXT; however, high resistance to clindamycin and erythromycin was still noticed in SCC*mec* types IV and V. In further antibiotic resistance gene characterization, our observations are in line with the findings of Teodoro, Mattos, Cavalcante, Pereira, and Santos (2012), in which the *erm*(A) gene was predominant in MRSA isolates and was correlated with SCC*mec* type III. In addition, they also found a relationship between *erm*(C) and SCC*mec *type IV. However, in our study, the *erm*(C) gene was found in SCC*mec* types II, IV and V indicating that this gene was not specifically related to any SCC*mec* type.

ST239‐III‐MRSA, which is a wellknown pathogen spread worldwide that has been associated with healthcare‐associated infections, was the predominant multidrug‐resistant clone found in our TCCs and surgical patients during the study period. Nevertheless, the dominant clone in our study, ST239‐III‐t037, which was reported by Chen, Liu, Jiang, Chen, and Wang (2010) as the most frequent clone in Beijing before 2000 and was gradually replaced by ST239‐III‐t030 was not found in our hospital. This incongruent result highlights the distribution of MRSA isolates due to geographical variations.

Biofilms play an important part in most human bacterial infections including infections associated with indwelling vascular grafts, peritoneal dialysis, and urinary catheters (Costerton, Stewart, & Greenberg, 1999). In a previous study, Yousefi et al. (2016) demonstrated that 27 out of 39 (69.2%) *S. aureus* samples from urinary tract infection (UTI) were biofilm producers. In contrast, all of the *S. aureus* isolates in this study were biofilm producers with strong adherence after 24 hr. Our results indicated the important role of biofilm formation in hemodialysis infection. Furthermore, we found that the biofilm formation ability of MRSA isolates correlated with SCC*mec* types. Our results are consistent with a previous study showing that SCC*mec* type III‐harboring strains showed a significantly higher ability to form biofilms compared with the strains harboring SCC*mec* type IV; however, no difference in biofilm formation with type II‐positive isolates was observed after 4 and 24 hr of incubation, which differs from the literature (William da Fonseca Batistao et al., 2016). The multidrug‐resistant SCC*mec* type III isolates produced significantly stronger biofilms than types IV and V suggesting that biofilm formation in MRSA isolates plays a role in antibiotic resistance in the hospital setting.

### 4.1 Study limitations

The major limitation of this investigation is that it was a nonrandomized study with a small number of patients. The sample size of 47 patients was small, and multiple isolates from single patients were analyzed, which may cause bias and overexaggeration of the results. Despite this limitation, we attempted to identify the relationship between the different types of vascular accesses and microbiology profiles along with biofilm formation abilities. This is the first prospective study that focused on *S. aureus *VAI and compared it with other hospitalized *S. aureus* surgical infections. The microbiology profiles can provide useful information regarding the management of patients undergoing hemodialysis.

## 5 CONCLUSIONS

In *S. aureus* dialysis vascular access infections, the isolates from TCCs had a higher percentage of methicillin resistance than the isolates from AVGs. MRSA in AVG–VAI was mostly SCC*mec* type IV. MRSA in TCC–VAI was similar to other hospitalized surgical infections and was predominantly SCC*mec* III, which had the strongest drug resistance and biofilm formation.

## CONFLICT OF INTERESTS

The authors have no conflict of interest to declare.

## AUTHORS CONTRIBUTION

Conceiving and designing the study: Chu C, Huang YK, and Lin CL; interpreting the data and writing manuscripts: Wong MY, Huang YK, Chu C, and Kao CC; obtaining funding: Huang YK, Tseng YH, and Tung CW. All authors read and approved the final manuscript.

## ETHICS STATEMENT

This study was approved by the Institutional Review Board (IRB) of the Chang Gung Memorial Hospital (IRB Nos: IRB101‐41888 and IRB‐8482B).

## DATA ACCESSIBILITY

The authors declare that the experimental data published in this paper are made accessible upon request for interested readers.
